# Insights into Abundant Rumen Ureolytic Bacterial Community Using Rumen Simulation System

**DOI:** 10.3389/fmicb.2016.01006

**Published:** 2016-06-28

**Authors:** Di Jin, Shengguo Zhao, Pengpeng Wang, Nan Zheng, Dengpan Bu, Yves Beckers, Jiaqi Wang

**Affiliations:** ^1^State Key Laboratory of Animal Nutrition, Institute of Animal Sciences, Chinese Academy of Agricultural SciencesBeijing, China; ^2^Animal Science Unit, Gembloux Agro-Bio Tech, University of LiègeGembloux, Belgium

**Keywords:** rumen, ureolytic bacteria, urea, acetohydroxamic acid, high-throughput sequencing

## Abstract

Urea, a non-protein nitrogen for dairy cows, is rapidly hydrolyzed to ammonia by urease produced by ureolytic bacteria in the rumen, and the ammonia is used as nitrogen for rumen bacterial growth. However, there is limited knowledge with regard to the ureolytic bacteria community in the rumen. To explore the ruminal ureolytic bacterial community, urea, or acetohydroxamic acid (AHA, an inhibitor of urea hydrolysis) were supplemented into the rumen simulation systems. The bacterial 16S rRNA genes were sequenced by Miseq high-throughput sequencing and used to reveal the ureoltyic bacteria by comparing different treatments. The results revealed that urea supplementation significantly increased the ammonia concentration, and AHA addition inhibited urea hydrolysis. Urea supplementation significantly increased the richness of bacterial community and the proportion of *ureC* genes. The composition of bacterial community following urea or AHA supplementation showed no significant difference compared to the groups without supplementation. The abundance of *Bacillus* and unclassified *Succinivibrionaceae* increased significantly following urea supplementation. *Pseudomonas, Haemophilus, Neisseria, Streptococcus*, and *Actinomyces* exhibited a positive response to urea supplementation and a negative response to AHA addition. Results retrieved from the NCBI protein database and publications confirmed that the representative bacteria in these genera mentioned above had urease genes or urease activities. Therefore, the rumen ureolytic bacteria were abundant in the genera of *Pseudomonas, Haemophilus, Neisseria, Streptococcus, Actinomyces, Bacillus*, and unclassified *Succinivibrionaceae*. Insights into abundant rumen ureolytic bacteria provide the regulation targets to mitigate urea hydrolysis and increase efficiency of urea nitrogen utilization in ruminants.

## Introduction

The use of urea in feeds of ruminants is increasing to reduce the supplementation of true protein and the costs of rations. The recommendations of urea would be for no more than 1% in the concentrate, ~135 g/cow daily (Kertz, 2010). In the rumen, ureolytic bacteria produce urease to hydrolyze urea to ammonia, which is subsequently used for the synthesis of amino acids and microbial protein. Normally, the rate of urea hydrolysis exceeds the rate of ammonia utilization, which leads to poor efficiency of urea utilization in the rumen and explosion of toxic ammonia in the blood (Patra, 2015). Acetohydroxamic acid (AHA), an inhibitor of urease activity that prevents the rapid hydrolysis of urea and consequent explosion of ammonia in rumen, is commonly applied in the rations of ruminants (Upadhyay, 2012).

Ureolytic bacteria play an important role in the hydrolysis of urea in the rumen. Previous studies have isolated some ureolytic bacteria from the rumen including *Succinovibrio dextrinosolvens, Treponema* sp., *Ruminococcus bromii, Butyrivibrio* sp., *Bifidobacterium* sp., *Prevotella ruminicola*, and *Peptostreptococcus productus* (Wozny et al., 1977). However, due to the difficulty in cultivating the rumen bacteria, those that have been isolated represent only 6.5% of the community (Kim et al., 2011). Thus, sequencing and phylogenetic analysis of 16S rRNA genes and functional genes have been extensively used in studies focused on members of the uncultured bacteria. By sequencing, ureolytic bacterial diversity has been observed in the environment including open oceans (Collier et al., 2009), groundwater (Gresham et al., 2007), sponges (Su et al., 2013), and soil (Singh et al., 2009). We have previously studied rumen ureolytic bacteria using a urease gene clone library, and found that ureolytic bacterial composition in the rumen was distinct from that in the environment (Zhao et al., 2015). Therefore, it is interesting and meaningful to explore the rumen ureolytic bacterial communities further.

Rumen simulation systems have been developed and used in the evaluation of feeds nutrients degradation and rumen fermentation manipulation in order to avoid the use of animals or decrease study costs (Hristov et al., 2012). We invented a dual-flow continuous rumen simulation system with real-time monitoring of pH, temperature, gas production, methane, and carbon dioxide concentration (Figure S1). We demonstrated that the conditions of microbial fermentation in the system were similar to those in the rumen of dairy cows (Shen et al., 2012), making it a powerful and practical tool for the study of rumen microbes or fermentation.

The objective of this study was to reveal abundant ureolytic bacterial community by high-throughput sequencing in a rumen simulation system when treated with an activator (urea) or inhibitor (AHA) of ureolytic bacteria.

## Materials and methods

### Experimental design and continuous cultivation

The rumen simulation system with eight fermenters were used in two replicated periods of 10 d each (7 d for adaptation and 3 d for sampling; Shen et al., 2012). The basic total mixed ration (TMR) was ground down to 1 mm for subsequent use. Fermenters were assigned to four treatments: U0_A0 (basic diet only), U0_A0.45 [basic diet plus AHA of 0.45 g/kg dry matter (DM)], U5_A0 (basic diet plus urea of 5 g/kg DM), U5_A0.45 (basic diet plus urea of 5 g/kg DM and AHA of 0.45 g/kg DM). Two fermenters were randomly assigned to each treatment in each period. A total of 40 g feed (DM based) was placed into each fermenter daily in two equal portions at 09:00 and 21:00. Urea and AHA were dissolved in artificial saliva (Weller and Pilgrim, 1974) and were added directly into the fermenters after each feeding. The basic diet (DM based) primarily consisted of alfalfa hay (17.72%), corn silage (17.50%), oaten hay (5.09%), cotton seed (5.61%), apple pulp (3.74%), sugar beet pulp (6.71%), and compound packet (40.95%). The compound packet provided the following per kg of diets: steam corn 180.39 g, soybean skin 55.84 g, soybean meal 64.43 g, extruded soybean 38.66 g, distillers dried grains with soluble (DDGS) 24.48 g, double-low rapeseed meal 25.77 g, Ca(HCO_3_)_2_ 2.58 g, CaCO_3_ 2.58 g, NaCl 3.44 g, and NaHCO_3_ 6.01 g (Table S1).

On the first day of each period, all fermenters were inoculated with ruminal fluid obtained from three rumen-fistulated cows fed the same TMR diet as used in the *in vitro* study. Animals involved in this study were cared for according to the principles of the Chinese Academy of Agricultural Sciences Animal Care and Use Committee (Beijing, China). Ruminal fluid was strained through four layers of cheesecloth and transferred to the laboratory in a sealed container. A total 500 mL of the strained ruminal fluid was added to each of the eight fermenters, which also contained 500 mL of artificial saliva. Anaerobic conditions were established by flushing the headspace of the fermenters with N_2_ at a rate of 20 mL min^−1^. The artificial saliva was continuously infused into the flasks. The temperature of the fermenters was maintained at 39°C by circulating water, and the fermenter content was stirred continuously at 25 rpm.

### Rumen fluid sampling and DNA extraction

During the last 3 days of each period, 3 mL of fermenter liquid was collected from each fermenter at 0, 2, 4, 6, 8, and 10 h after morning feeding. Collected samples were stored at −80°C for detection of ammonia nitrogen (NH_3_-N) and urea nitrogen (urea-N) concentrations. The NH_3_-N concentration was determined using the method based on the Berthelot (phenol–hypochlorite) reaction (Broderick and Kang, 1980). Urea nitrogen (urea-N) concentration was determined using the diacetyl monoxime method with a commercial kit (Nanjing Jiancheng Co., Nanjing, China). Rumen fluid collected at 2 h was used to extract microbial DNA with a cetyl trimethylammonium bromide (CTAB) plus bead beating method (Minas et al., 2011). Extracted DNA was assessed by agarose gel (1%) electrophoresis and quantified using a Nanodrop™ spectrometer (Thermo Scientific, Waltham, MA, USA).

### Quantitative PCR of urease and 16S rRNA genes

The urease alpha subunit encoding gene (*ureC)* primers UreC-F (5′-TGGGCCTTAAAATHCAYGARGAYTGGG-3′) and UreC-R (5′-SGGTGGTGGCACACCATNANCATRTC-3) were used to quantify the *ureC* gene copies (Reed, 2001). 16S rRNA gene of total bacteria were quantified using 338-F (5′-ACTCCTACGGGAGGCAGCAG-3′) and 533-R (5′-TTA CCGCGGCTGCTGGCAC -3′) as primers (Huse et al., 2008). The assays were performed in an iQ™5 Multicolor Real-Time PCR Detection System (Bio-Rad, Hercules, CA, USA) using SYBR® Premix Ex Taq™ II (Takara, Dalian, China). Standard curves were generated using plasmids DNA cloned with *ureC* gene or 16S rRNA gene (Figure S2). Copy number of *ureC* gene or 16S rRNA gene in per ng of DNA was determined by relating the CT value to the standard curves. The proportion of *ureC* gene copies was calculated as the ratio of *ureC* gene copies to total 16S rRNA gene copies. The detailed qPCR protocols were provided in the Supplementary Material. The proportion of *ureC* gene copies in each treatment were shown in a boxplot constructed using R (R Core Team, 2013).

### Bacterial 16S rRNA genes amplification and illumina sequencing

Microbial DNA was used as a template for amplification of partial 16S rDNA sequence using the universal bacterial primers 515F (5′-GTGCCAGCMGCCGCGGTAA-3′) and 806R (5′- GGACTACHVGGGTWTCTAAT-3′; Nelson et al., 2014) with both primers tagged with unique barcode sequences for each sample. All polymerase chain reactions (PCRs) were carried out in 50 μL reactions with 0.5 μL of PrimeSTAR® HS DNA Polymerase (TaKaRa, Dalian, China), 10 μL 5 × PrimeSTAR Buffer (plus Mg^2+^) (TaKaRa), 0.2 μM of the forward and reverse primers, 200 μM dNTP (TaKaRa), and 100 ng microbial DNA. Thermal cycling consisted of initial denaturation at 98°C for 1 min, followed by 30 cycles of denaturation at 98°C for 10 s, annealing at 50°C for 30 s, and elongation at 72°C for 60 s, and a final elongation at 72°C for 5 min. Unique bands were identified using agarose gel (2%) electrophoresis of PCR amplicons (Figure S3). The bands were cut and purified with a QIAGEN MinElute PCR Purification Kit (Qiagen, Valencia, CA, USA). Amplicon libraries were generated using NEB Next® Ultra™ DNA Library Prep Kit for Illumina (New England Biolabs, Ipswich, MA, USA) following the manufacturer's recommendations, with the addition of index codes. Library quality was assessed on the Qubit® 2.0 Fluorometer (Thermo Scientific) and Agilent Bioanalyzer 2100 system. The library was sequenced on an Illumina MiSeq platform (2 × 250 bp).

### Sequencing data processing and analysis

Paired-end reads were merged using FLASH (Magoè and Salzberg, 2011). Merged reads were assigned to each sample based on the unique barcode, after which the barcodes and primers were removed. The quality of raw reads was checked, and reads were truncated at any site of >3 sequential bases receiving a quality score of < Q20, and reads with < 75% (of total read length) consecutive high quality base calls were removed (Caporaso et al., 2010; Bokulich et al., 2013). Chimeric sequences were detected and removed using UCHIME (Haas et al., 2011). Operational taxonomic units (OTU) were generated by aligning the reads to the GreenGenes database released in May 2013 (DeSantis et al., 2006) and clustered at 97% sequence identity using the PyNAST tool (Caporaso et al., 2010) and the UCLUST algorithm (Edgar et al., 2011). The OTUs were filtered based on the total observation count of an OTU < 10 and the number of samples in an OTU < 2 in QIIME (Caporaso et al., 2010). The OTUs were further assigned to taxa using the RDP classifier (Wang et al., 2007). The OTU table was rarified for alpha diversity analysis. Simpson, Shannon, Chao1, and the PD_whole_tree index were calculated for each sample. Good's coverage was used to estimate the percentage of the total species that were sequenced in each sample (Caporaso et al., 2010). QIIME was used to calculate the weighted UniFrac distances, which are phylogenetic measures of beta diversity. The weighted UniFrac distance was used for Principal Coordinate Analysis (PCoA; Lozupone et al., 2007). The significance of grouping in the PCoA plot was tested by analysis of similarity (ANOSIM) in QIIME with 999 permutations (R Core Team, 2013; Mahnert et al., 2015). The relative abundance of bacteria was expressed as the percentage. The potential ureolytic bacteria were selected using the criterion that their abundance increased with urea treatment and decreased with AHA treatment. The urease alpha subunit sequences of representative species from potential ureolytic bacteria were checked against the NCBI protein database and the urease activities of these bacteria were verified by published studies.

### Statistical analysis

Urea-N, ammonia, proportion of *ureC* gene copies, bacterial abundance, and diversity index were statistical analyzed using the SAS MIXED procedure (SAS Institute, Inc, Cary, NC) as shown in the following model: Y_*ijk*_ = μ + a_*i*_ + b_*j*_ + ab_*ij*_ + *e*_*ijk*_, where Y_*ijk*_ is the dependent variable, μ is the overall mean, a_*i*_ is the effect of urea treatment i, b_j_ is the effect of AHA treatment j, ab_ij_ is the interaction between a_i_ and b_j_ (Both factors and their interaction are considered fixed effects), and *e*_*ijk*_ is the residual, assumed to be normally distributed. Data of bacterial abundance were transformed to log_10_ (n+1) if necessary to ensure normal distribution. Mean separation was conducted by using Fisher's least significant difference test. Differences were declared significant at *P* < 0.05. Tukey's test was used to determine where the differences occurred.

### Nucleotide sequence accession number

All the raw sequences after assembling and filtering were submitted to the NCBI Sequence Read Archive (SRA; http://www.ncbi.nlm.nih.gov/Traces/sra/), under accession number SRP074113.

## Results

### Changes of urea, ammonia concentrations, and proportion of *ureC* genes

**Figure 1:**
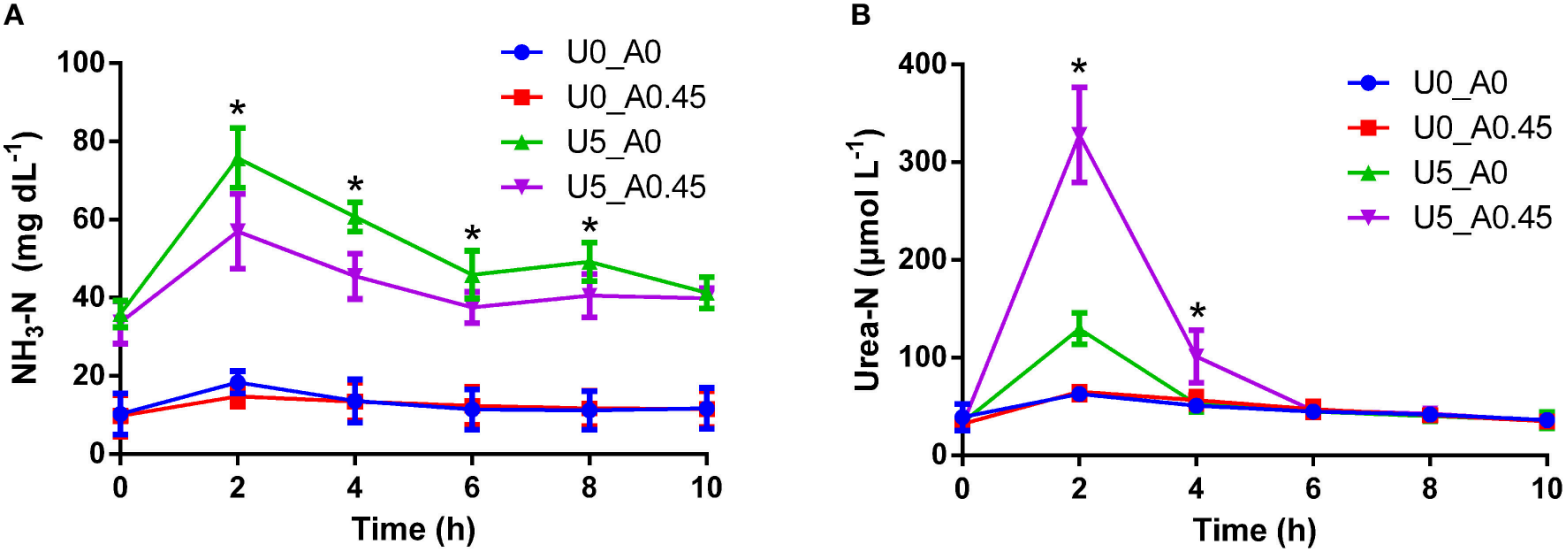
**Changes of NH_**3**_-N and urea-N concentrations induced by urea and AHA supplementation**. **(A)** Changes of NH_3_-N concentration. **(B)** Changes of urea-N concentration. U0_A0, basic diet only; U0_A0.45, basic diet plus AHA of 0.45 g/kg DM; U5_A0, basic diet plus urea of 5 g/kg DM; U5_A0.45, basic diet plus urea of 5 g/kg DM and AHA of 0.45 g/kg DM. ^*^Means values in group U5_A0 was significantly different from that in group U5_A0.45 (*P* < 0.05).

**Figure 2:**
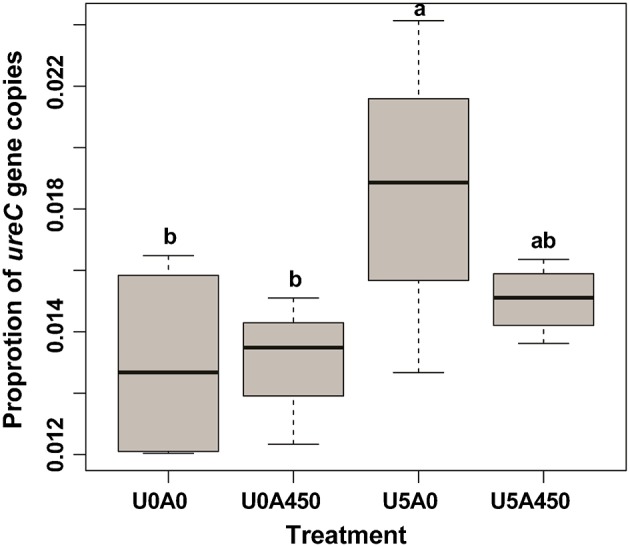
**Changes in the proportion of ***ureC*** gene copies induced by urea and AHA supplementation**. The proportion of *ureC* gene copies was calculated as the ratio of *ureC* gene copies to total 16S rRNA gene copies. U0_A0, basic diet only; U0_A0.45, basic diet plus AHA of 0.45 g/kg DM; U5_A0, basic diet plus urea of 5 g/kg DM; U5_A0.45, basic diet plus urea of 5 g/kg DM and AHA of 0.45 g/kg DM. ^a,b^Different letters for different treatments indicate statistically significant differences (*P* < 0.05).

The urea-N concentrations in the two urea treated groups were higher (*P* < 0.01) than the other two groups at 2 h after morning feeding (Figure 1). In the two urea treated groups, Group U5_A0.45 exhibited a higher (*P* < 0.01) urea concentration than group U5_A0, indicating a decreased urea hydrolysis rate with AHA inhibition (Figure 1). The NH_3_-N concentrations of all four treatments showed a peak value after fermentation for 2 h. Urea supplementation significantly increased (*P* < 0.01) NH_3_-N concentration during whole sampling period, while in the two urea-treated groups, AHA addition also decreased NH_3_-N concentration significantly (*P* < 0.01). Two hours after the morning feeding, the proportion of *ureC* genes was higher (*P* < 0.05) in urea-treated groups than in non-urea treated groups. The addition of AHA did not have a significant effect on the proportion of *ureC* genes (Figure 2).

### Changes of ureolytic bacterial diversity

**Figure 3:**
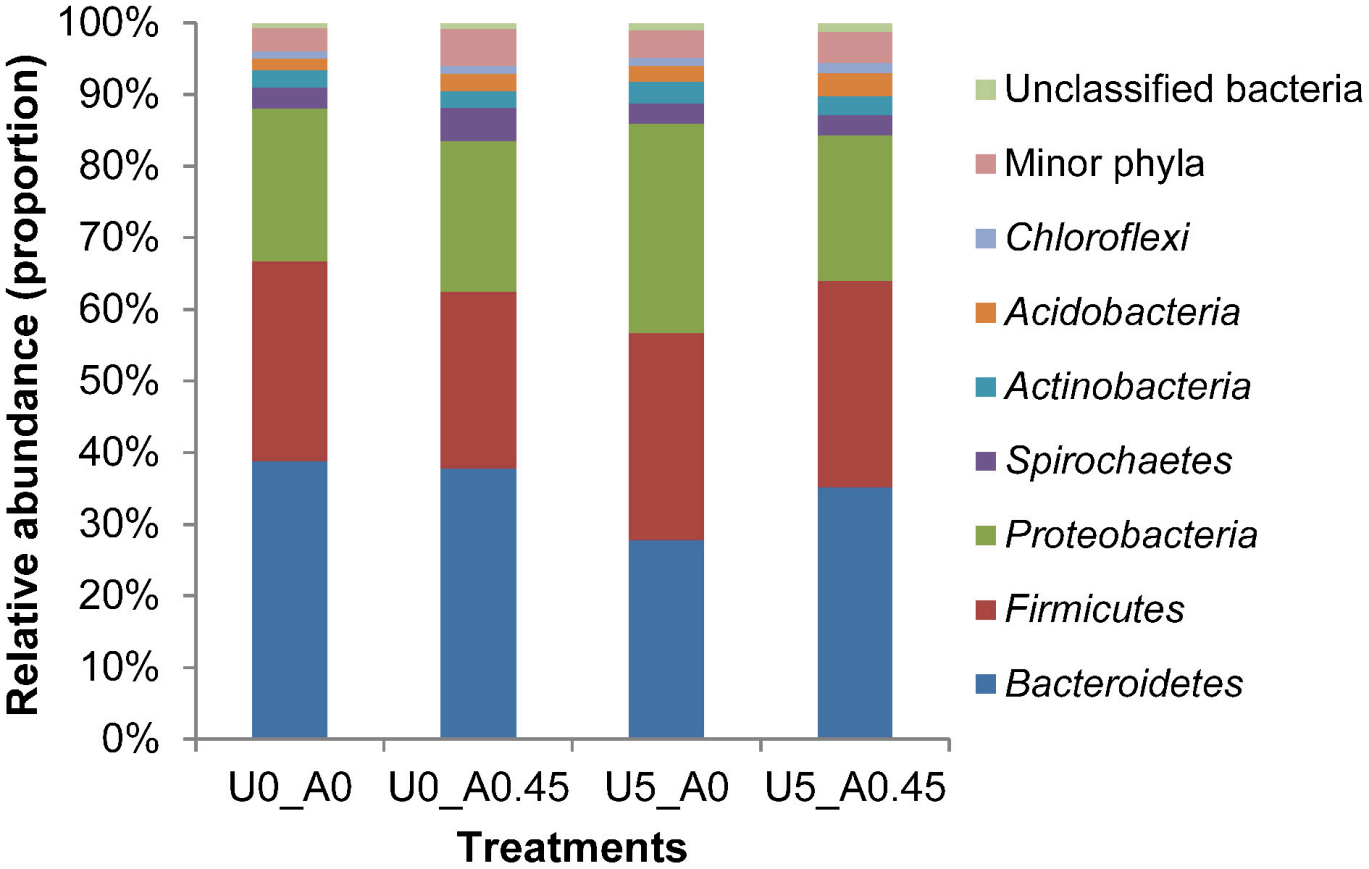
**Composition of the most predominant bacterial phyla in the rumen**. U0_A0, basic diet only; U0_A0.45, basic diet plus AHA of 0.45 g/kg DM; U5_A0, basic diet plus urea of 5 g/kg DM; U5_A0.45, basic diet plus urea of 5g/kg DM and AHA of 0.45 g/kg DM.

**Figure 4:**
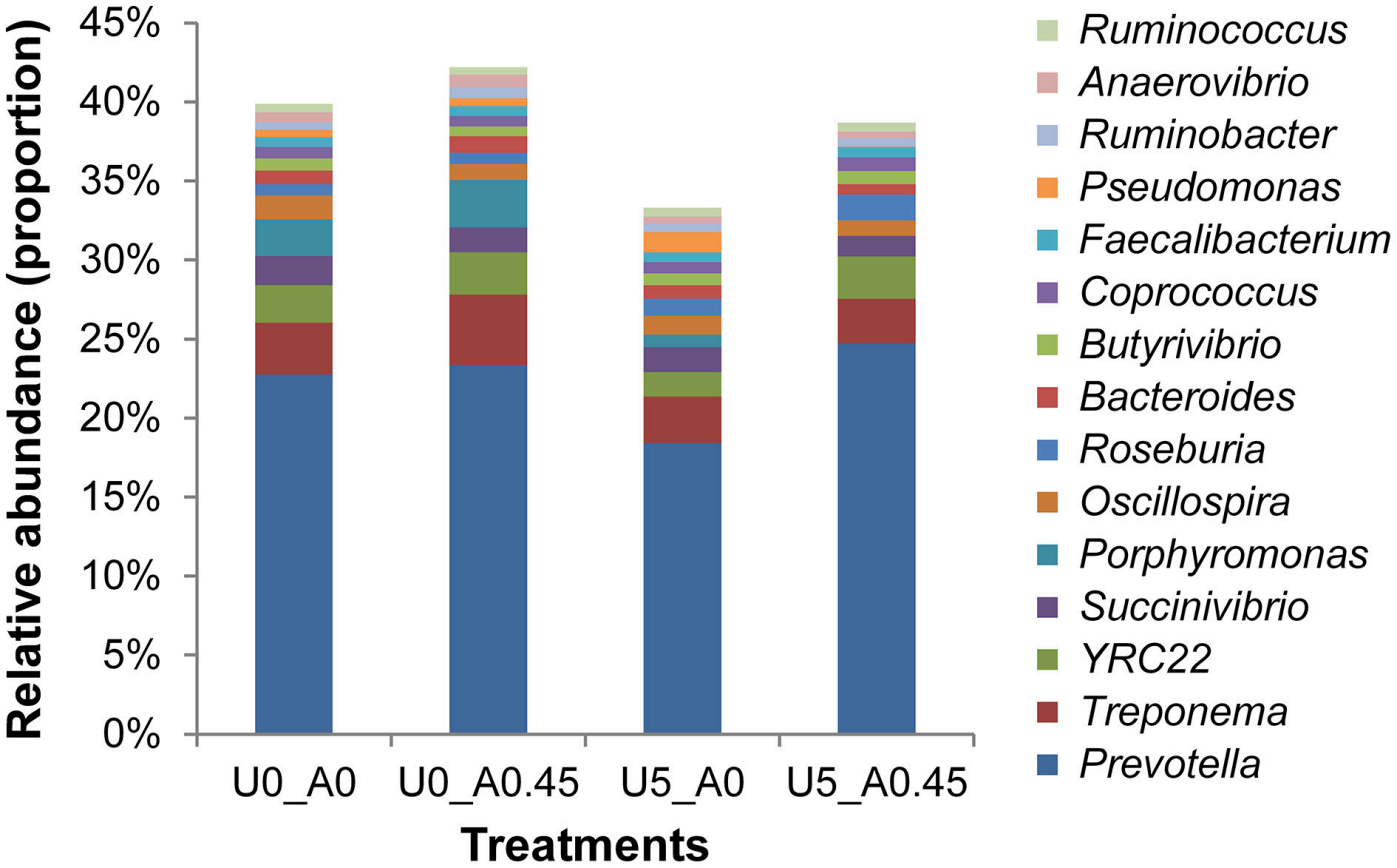
**Composition of the most predominant bacterial genera in the rumen**. U0_A0, basic diet only; U0_A0.45, basic diet plus AHA of 0.45 g/kg DM; U5_A0, basic diet plus urea of 5 g/kg DM; U5_A0.45, basic diet plus urea of 5 g/kg DM and AHA of 0.45 g/kg DM. The top 15 abundant bacteria genera were shown and the others were not shown. Other genera accounted for 60.11% in group U0_A0, 57.81% in group U0_A0.45, 66.68% in group U5_A0, and 61.31% in group U5_A0.45.

A total of 2,105,448 merged sequences were acquired from 16 samples, and 1,672,529 high-quality sequences, with an average read length of 253 bases were obtained. After removing chimeric sequences, the remaining 1,603,997 sequences were used to generate OTUs with 97% sequence similarity across all samples. The OTU table was filtered, leaving 5075 OTUs for subsequent analysis. Collectively, 24 bacterial phyla were identified. *Bacteroidetes, Firmicutes*, and *Proteobacteria* were the three predominant phyla, representing 35, 28, and 23% of all sequences, respectively (Figure 3). Genera that were each represented by ≥ 0.1% of the total sequences in at least 1 of the 16 samples were selected for further analysis. The 10 predominant genera were *Prevotella, Treponema, YRC22, Succinivibri*o, *Porphyromonas, Oscillospira, Roseburia, Bacteroides, Butyrivibrio*, and *Coprococcus* (Figure 4).

**Figure 5:**
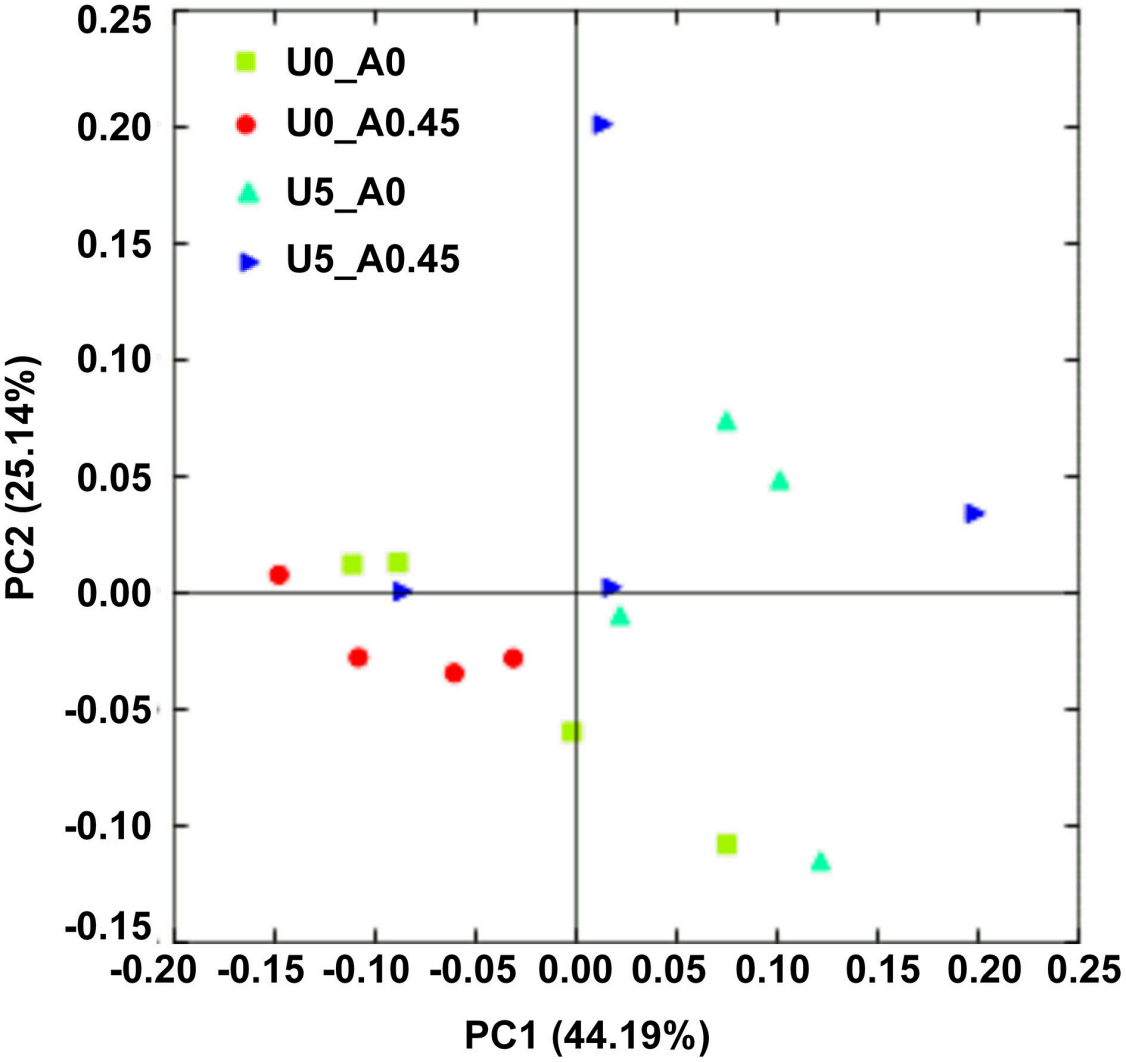
**Principal coordinate analysis (PCoA) of the rumen bacterial community**. The principal coordinate analysis is based on the weighted UniFrac distances between the microbiome profiles. U0_A0, basic diet only; U0_A0.45, basic diet plus AHA of 0.45 g/kg DM; U5_A0, basic diet plus urea of 5 g/kg DM; U5_A0.45, basic diet plus urea of 5 g/kg DM and AHA of 0.45 g/kg DM.

After rarefaction, 9000 sequences per sample were used for diversity analysis. Alpha bacterial diversity was presented in Table 1. Group U5_A0 had the highest Chao 1 and PD_whole_tree estimates, followed by groups U5_A0.45, U0_A0.45, and U0_A0. No significant differences were observed among the four groups based on the results of the Simpson and Shannon diversity index. PCoA analysis of overall diversity based on the unweighted UniFrac metrics was performed to compare the four treatments (Figure 5). ANOSIM (cutoff = 0.01) showed no significant differences in bacterial community composition between treatments U0_A0 and U0_A0.45 (*R* = −0.198, *P* = 0.925) or between treatments U5_A0 and U5_A0.45 (*R* = −0.135, *P* = 0.888). A tendency of difference was found between treatments U0_A0 and U5_A0 (*R* = 0.323, *P* = 0.091). Principal Coordinate 1 and 2 accounted for 44.19 and 25.14% of the total variation, respectively.

**Table 1:** **Alpha diversity index of rumen bacteria among all treatments**.

**Indices**	**U0**		**U5**		**SEM**	***P-*****value**		
	**A0**	**A0.45**	**A0**	**A0.45**		**Urea**	**AHA**	Urea^*^AHA
Observed_species	1442	1496	1557	1563	25	0.11	0.54	0.62
Good's coverage	0.914^a^	0.911^ab^	0.905^b^	0.906^b^	0.002	0.02	0.62	0.46
PD_whole_tree	106^b^	109^ab^	111^a^	109^ab^	0.89	0.09	0.70	0.11
Chao 1	2860^c^	2942^bc^	3142^a^	3043^ab^	43	0.01	0.85	0.11
Shannon	7.59	7.73	7.77	7.59	0.08	0.92	0.92	0.46
Simpson	0.96	0.96	0.97	0.95	0.01	0.89	0.50	0.36

a−c *Mean values within a row with different letters differ significantly (P < 0.05). SEM, standard error of the mean. U0, basic diet without urea; U5, basic diet plus urea of 5 g/kg DM; A0, basic diet without AHA; A0.45, basic diet plus AHA of 0.45 g/kg DM*.

### Changes of the relative abundance of ureolytic bacteria

At the phylum level, the group treated with urea only had the highest proportion of *Proteobacteria* and *Actinobacteria*, and the lowest proportion of *Bacteroidetes* compared with the other three groups (Figure 3). Both of the two urea-treated groups had relatively high proportions of *Acidobacteria* and low proportions of *Spirochaetes* compared with the other two groups. In addition, the two urea-treated groups had higher percentages of unclassified bacteria than the other two groups. At the genus level, the relative abundance represented by ≥0.1% of the total sequences in at least one of the whole samples were further analyzed (Table 2). *Pseudomonas* (1.25%) from *Proteobacteria* and *Streptococcus* (1.00%) from *Firmicutes* were more predominant in group U5_A0 compared to the other three groups (*P* < 0.01). *Haemophilus* and *Neisseria* from *Proteobacteria*, and *Actinomyces* from *Actinobacteria* were the most abundant in the U5_A0 group compared with the other three groups (*P* < 0.05). The relative abundance of *Bacillus* from *Firmicutes* and unclassified *Succinivibrionaceae* were higher in the two urea-treated groups compared with the other two groups (*P* < 0.01). According to the results retrieved from the NCBI protein database and reported in previous studies, the representative species from *Pseudomonas, Haemophilus, Streptococcus, Neisseria, Bacillus, Actinomyces*, and unclassified *Succinivibrionaceae* were identified as containing urease genes and having urease activity (Table 3).

**Table 2:** **Bacterial genera that accounted for ≥0.1% of the total sequences in at least one of the samples with significant variation under different treatments (abundance of the genera was expressed as %)**.

**Taxa (family and genus within each phylum)**		**U0**		**U5**		**SEM**	***P-*****value**		
		**A0**	**A0.45**	**A0**	**A0.45**		**Urea**	**AHA**	Urea^*^AHA
*Bacteroidetes*	*Porphyromonadaceae; Paludibacter*	0.13^ab^	0.20^a^	0.02^b^	0.00^b^	0.0003	0.0175	0.6091	0.3539
	*Chitinophagaceae;* unclassified genus	0.15^b^	0.19^a^	0.16^ab^	0.11^b^	0.0001	0.0361	0.5310	0.0080
*Proteobacteria*	*Succinivibrionaceae;* others	8.17^a^	6.76^ab^	5.06^b^	4.04^b^	0.0063	0.0067	0.0989	0.7615
	*Succinivibrionaceae;* unclassified genus	1.11^b^	1.05^b^	6.05^a^	4.38^a^	0.0083	0.0008	0.1279	0.1488
	*Pseudomonadaceae; Pseudomonas*	0.49^b^	0.50^b^	1.25^a^	0.05^b^	0.0020	0.2671	0.0075	0.0071
	*Pasteurellaceae; Haemophilus*	0.02^b^	0.03^b^	1.92^a^	0.00^b^	0.0005	< 0.0001	< 0.0001	< 0.0001
	*Neisseriaceae; Neisseria*	0.05^b^	0.02^b^	0.66^a^	0.00^b^	0.0003	0.0193	0.0111	0.0153
	*Desulfobulbaceae; Desulfobulbus*	0.21^a^	0.14^ab^	0.02^ab^	0.01^b^	0.0004	0.0360	0.4308	0.6472
	*Campylobacteraceae; Campylobacter*	0.11^ab^	0.13^a^	0.04^ab^	0.00^b^	0.0002	0.0400	0.7626	0.3891
	*Moraxellaceae; Acinetobacter*	0.04^ab^	0.02^b^	0.07^ab^	1.10^a^	0.0001	0.0516	0.5991	0.2099
*Firmicutes*	*Clostridiaceae;* unclassified genus	8.04^ab^	6.10^b^	8.40^ab^	9.32^a^	0.0051	0.0483	0.4704	0.0888
	*Acidaminobacteraceae;* unclassified genus	0.15^a^	0.16^a^	0.04^b^	0.00^b^	0.0003	0.0182	0.6995	0.5929
	*Lachnospiraceae; Roseburia*	0.72^b^	0.73^b^	1.08^b^	1.64^a^	0.0016	0.0051	0.0634	0.0731
	*Lachnospiraceae; Lachnospira*	0.20^b^	0.16^b^	0.23^ab^	0.37^a^	0.0003	0.0338	0.2710	0.0818
	*Veillonellaceae; Anaerovibrio*	0.63^ab^	0.75^a^	0.42^b^	0.43^b^	0.0006	0.0297	0.4459	0.5064
	*Veillonellaceae; Veillonella*	0.00^b^	0.01^b^	0.53^a^	0.00^b^	0.0026	0.0096	0.0093	0.0090
	*Peptostreptococcaceae; Filifactor*	0.81^a^	0.69^a^	0.25^b^	0.00^b^	0.0014	0.0041	0.1435	0.5827
	*Streptococcaceae; Streptococcus*	0.17^b^	0.31^b^	1.00^a^	0.14^b^	0.0002	0.0135	0.0103	0.0030
	*Bacillaceae; Bacillus*	0.06^c^	0.09^bc^	0.13^a^	0.17^a^	0.0051	0.0062	0.0858	0.7493
*Actinobacteria*	*Micrococcaceae; Arthrobacter*	0.07^b^	0.09^ab^	0.11^a^	0.03^b^	0.0001	0.5271	0.0651	0.0076
	*Actinomycetaceae; Actinomyces*	0.03^b^	0.04^b^	0.18^a^	0.00^b^	0.0026	0.1007	0.0286	0.0199

a−c *Means values within a row with different letters differ significantly (P < 0.05). SEM, standard error of the mean. U0, basic diet without urea; U5, basic diet plus urea of 5 g/kg DM; A0, basic diet without AHA; A0.45, basic diet plus AHA of 0.45 g/kg DM*.

**Table 3:** **Urease gene and enzyme activity of selected genera containing ureolytic bacteria in rumen**.

**Genus**	**Representative species**	**Urease gene (Alpha subunitaccession in NCBI)**	**Urease activity (References)**
			
Unclassified *Succinivibrionaceae*	*Succinivibrionaceae* WG-1	+ (WP 010457200)	+ (Pope et al., 2011)
*Pseudomonas*	*Pseudomonas aeruginosa* BG	+ (KM657955	+ (Goswami et al., 2015)
	*Pseudomonas fluorescens*	+ (KPU59664)	+ (Jyothi and Umamahe, 2013)
*Haemophilus*	*Haemophilus influenza* Rd	+ (KMZ31254)	+ (McCrea et al., 2008)
	*Haemophilus haemolyticus*	+ (WP 005644404)	+ (McCrea et al., 2008)
*Streptococcus*	*Streptococcus thermophiles*	+ (KPL38034)	+ (Zotta et al., 2008)
	*Streptococcus salivarius* 57.I	+ (AEJ54136)	+ (Chen et al., 2000)
*Neisseria*	*Neisseria sp*. KH1503	+ (KLT73764)	+ (Sakai et al., 1996)
*Bacillus*	*Bacillus cereus*	+ (AAS42567)	+ (Rasko et al., 2004)
	*Bacillus pasteurii*	+ (1S3T_C)	+ (Benini et al., 2000)
*Actinomyces*	*Actinomyces naeslundii*	+ (AAD13732)	+ (Morou-Bermudez and Burne, 2000)
	*Actinomyces johnsonii*	+ (WP 021610181)	+ (Schaal and Yassin, 2015)

*+Positive urease genes or enzyme activity*.

## Discussion

In the rumen, urea is a source of nitrogen for the growth of ureolytic bacteria. AHA, an inhibitor of urease, inhibits urea usage by ureolytic bacteria, and results in insufficient nitrogen source for bacterial growth. In this study, we used urea and AHA to promote or inhibit the growth of rumen ureolytic bacteria, respectively. We observed that AHA is a useful inhibitor for slowing down the hydrolysis of urea within the rumen fluid. This is consistent with previously published studies *in vivo* (Jones and Milligan, 1975; Makkar et al., 1981).

Urea supplementation significantly increased bacterial community richness and the number of bacterial species. AHA supplementation resulted in no changes of richness and diversity of bacterial community. The proportion of urease gene copies was served as a proxy to observe changes in the proportion of ureolytic bacteria. Urea supplementation significantly increased the proportion of ureolytic bacteria, which suggested that urea stimulated the growth of rumen ureolytic bacteria. In addition, ANOSIM revealed that the composition of the entire bacterial community in urea-treated groups showed a trend of difference from those in non-urea treated groups (*P* < 0.10). Changes of the bacterial community in response to urea treatment were possibly related to urease activity and the production of ammonia. Kim et al. (2014) found that urease genes and enzyme activities were regulated by the level of ammonia in ruminal cellulytic bacteria *Ruminococcus albus* 8. The lack of a significant effect by AHA on the diversity of the rumen bacterial community may be due to microbial adaption of AHA. Previous studies found that rumen microbe could adapt to chronic AHA supplementation, while AHA was capable of short-term inhibition of urease activity in the rumen (Zhang et al., 2001).

Across the four groups, three phyla (*Bacteroidetes, Firmicutes*, and *Proteobacteria*) were predominant. Similar to our results previously published studies have reported that the distribution of phylotypes of rumen bacterial communities fell predominantly into these three phyla (Hook et al., 2011; Wu et al., 2012; Zhang et al., 2014). The bacterial community from our *in vitro* simulation system was thus similar to the communities observed *in vivo*. The group treated with urea only had the highest proportion of *Proteobacteria* and the lowest proportion of *Bacteroidetes*. In accordance, Collier et al. (2009) investigated the diversity of ureolytic microorganisms in open ocean and estuarine planktonic communities, and found that ureolytic microorganisms were most commonly found in *Proteobacteria* and rare in *Bacteroidetes*.

*Bacillus* was in higher abundance in the two groups supplemented with urea, indicating it was more responsive to urea. *Bacillus* spp. in the rumen is able to degrade hemicellulose, and produce polysaccharidases and glycoside hydrolases to utilize polysaccharide (Williams and Withers, 1983). *B*. *pasteurii, B. lentus*, and *B*. *cereus* have proven to be ureolytic bacteria (Benini et al., 2000; Rasko et al., 2004; Sarda et al., 2009), and the urease activity of *B*. *pasteurii* is inhibited by AHA (Benini et al., 2000). The unclassified *Succinivibrionaceae* was also observed at a higher relative abundance in the two urea-treated groups. In the rumen, *Succinivibrionaceae* is very common and important for degradation of starch, pectin, and dextrin to succinate and propionate (Santos and Thompson, 2014). *Succinivibrionaceae* WG-1 isolated from the foregut of tammar wallaby produced urease for urea catabolism (Pope et al., 2011). Several isolates of *S*. *dextrinosolvens* from the rumen were also shown to have urease activity (Wozny et al., 1977).

*Pseudomonas* and *Streptococcus* were both relatively more abundant in the group treated with urea only, but these bacteria had lower abundance in AHA-treated groups. These results confirmed the urea stimulating and AHA inhibiting effects on the microbial community. Several species of *Pseudomonas* and *Streptococcus* are able to hydrolyze cellulose (Lynd et al., 2002; Oyeleke and Okusanmi, 2008). In the genus *Pseudomonas*, species such as *P*. *fluorescens* (isolated from soil) and *P. aeruginosa* (isolated from ocean) possess urease activity (Jyothi and Umamahe, 2013; Goswami et al., 2015). In addition, two *Streptococcal* species, *S. thermophiles* and *S*. *salivarius*, also produce urease (Chen et al., 2000; Zotta et al., 2008). Kakimoto et al. (1989) assayed about 16,000 isolates from animal feces and intestines for production of acid urease, and found 370 urease-positive strains belonging to the genus *Streptococcus*. This is consistent with the results of our study in which *Streptococcus* were found in higher abundance in response to urea supplementation.

The relative abundance of genera *Haemophilus, Neisseria*, and *Actinomyces* increased in response to urea and decrease in response to AHA supplementation. The members of *Haemophilus* ferment glucose (Kilian, 2015), and *H*. *haemolyticus* and *H*. *influenzae* Rd have urease activity (McCrea et al., 2008). The *H*. *somnus* strains of ruminants have varying urea hydrolysis ability (Garcia-Delgado et al., 1977). *Neisseria*, a Gram-negative aerobic cocci, produces acid from different types of sugars, and some species are disease-causing (Marri et al., 2010). *N*. *sicca* strains SB and SC isolated from soil have proven to be urease positive (Sakai et al., 1996). *Neisseria* had a higher proportion in groups treated with urea, suggesting the potential of bacterial species in the rumen to have urea hydrolysis activity. *Actinobacteria*, a group of Gram-positive bacteria, represent up to 3.00% of the total rumen bacteria (Pandya et al., 2010; Šul'ák et al., 2012). Some strains of *A*. *meyeri, A*. *radicidentis*, and *A*. *johnsonii* are known to have urease activity (Schaal and Yassin, 2015), and *A*. *naeslundii* had urease gene and activity (Morou-Bermudez and Burne, 1999, 2000). However, An et al. (2006) described a novel species, *Actinomyces ruminicola* sp., from cattle rumen, was unable to hydrolyze urea. So it needs to be verified for ureolytic activity of different *Actinomyces* species.

## Conclusion

The composition of bacterial community following urea or AHA supplementation treatment showed no significant difference compared to the groups without supplementation. In the rumen, the ureolytic bacteria were abundant in the genera including *Pseudomonas, Streptococcus, Haemophilus, Bacillus, Neisseria, Actinomyces*, and unclassified *Succinivibrionaceae*. The insights into abundant ureolytic bacteria provide the basis for designing strategies to efficiently manipulate the bacterial community or function and improve urea utilization in ruminant production.

## Author contributions

JW, DB, and SZ designed the experiments. DJ and PW performed the experiments. SZ and DJ analyzed the data. DJ wrote the paper. SZ, NZ, and YB revised the paper. All authors agree to be accountable for all aspects of the work.

### Conflict of interest statement

The authors declare that the research was conducted in the absence of any commercial or financial relationships that could be construed as a potential conflict of interest.

## References

[B1] An D. Cai S. Dong X. (2006). *Actinomyces ruminicola* sp. nov., isolated from cattle rumen. Int. J. Syst. Evol. Microbiol. 56, 2043–2048. 10.1099/ijs.0.64059-0 16957097

[B2] Benini S. Rypniewski W. R. Wilson K. S. Miletti S. Ciurli S. Mangani S. (2000). The complex of *Bacillus pasteurii* urease with acetohydroxamate anion from X-ray data at 1.55 Å resolution. J. Biol. Inorg. Chem. 5, 110–118. 10.1007/s007750050014 10766443

[B3] Bokulich N. A. Subramanian S. Faith J. J. Gevers D. Gordon J. I. Knight. (2013). Quality-filtering vastly improves diversity estimates from Illumina amplicon sequencing. Nat. Meth. 10, 57–59. 10.1038/nmeth.2276 23202435 PMC3531572

[B4] Broderick G. A. Kang J. H. (1980). Automated simultaneous determination of ammonia and total amino acids in ruminal fluid and *in vitro* media. J. Dairy Sci. 63, 64–75. 10.3168/jds.S0022-0302(80)82888-8 7372898

[B5] Caporaso J. G. Kuczynski J. Stombaugh J. Bittinger K. Bushman F. D. (2010). QIIME allows analysis of highthroughput community sequencing data. Nat. Methods 7, 336–336. 10.1038/nmeth.f.303 20383131 PMC3156573

[B6] Chen Y.-Y. M. Weaver C. A. Burne R. A. (2000). Dual functions of *Streptococcus salivarius* urease. J. Bacteriol. 182, 4667–4669. 10.1128/JB.182.16.4667-4669.2000 10913107 PMC94645

[B7] Collier J. L. Baker K. M. Bell S. L. (2009). Diversity of urea-degrading microorganisms in open-ocean and estuarine planktonic communities. Environ. Microbiol. 11, 3118–3131. 10.1111/j.1462-2920.2009.02016.x 19659552

[B8] DeSantis T. Z. Hugenholtz P. Larsen N. Rojas M. Brodie E. L. Keller K.. (2006). Greengenes, a chimera-checked 16S rRNA gene database and workbench compatible with ARB. Appl. Environ. Microbiol. 72, 5069–5072. 10.1128/AEM.03006-05 16820507 PMC1489311

[B9] Edgar R. C. Haas B. J. Clemente J. C. Quince C. Knight R. (2011). UCHIME improves sensitivity and speed of chimera detection. Bioinformatics 27, 2194–2200. 10.1093/bioinformatics/btr381 21700674 PMC3150044

[B10] Garcia-Delgado G. Little P. Barnum D. (1977). A comparison of various haemophilus somnus strains. Can. J. Comp. Med. 41, 380. 922555 PMC1277736

[B11] Goswami D. Patel K. Parmar S. Vaghela H. Muley N. Dhandhukia P. (2015). Elucidating multifaceted urease producing marine *Pseudomonas aeruginosa* BG as a cogent PGPR and bio-control agent. Plant Growth Regul. 75, 253–263. 10.1007/s10725-014-9949-1

[B12] Gresham T. L. T. Sheridan P. P. Watwood M. E. Fujita Y. Colwell F. S. (2007). Design and validation ofurec-based primers for groundwater detection of urea-hydrolyzing bacteria. Geomicrobiol. J. 24, 353–364. 10.1080/01490450701459283

[B13] Haas B. J. Gevers D. Earl A. M. Feldgarden M. Ward D. V. Giannoukos G.. (2011). Chimeric 16S rRNA sequence formation and detection in Sanger and 454-pyrosequenced PCR amplicons. Genome Res. 21, 494–504. 10.1101/gr.112730.110 21212162 PMC3044863

[B14] Hook S. E. Steele M. A. Northwood K. S. Dijkstra J. France J. Wright A. D.. (2011). Impact of subacute ruminal acidosis (SARA) adaptation and recovery on the density and diversity of bacteria in the rumen of dairy cows. FEMS Microbiol. Ecol. 78, 275–284. 10.1111/j.1574-6941.2011.01154.x 21692816

[B15] Hristov A. N. Lee C. Hristova R. Huhtanen P. Firkins J. L. (2012). A meta-analysis of variability in continuous-culture ruminal fermentation and digestibility data. J. Dairy Sci. 95, 5299–5307. 10.3168/jds.2012-5533 22916935

[B16] Huse S. M. Dethlefsen L. Huber J. A. Welch D. M. Relman D. A. Sogin M. L. (2008). Exploring microbial diversity and taxonomy using SSU rRNA hypervariable tag sequencing. PLoS Genet. 4:e1000255. 10.1371/journal.pgen.1000255 19023400 PMC2577301

[B17] Jones G. A. Milligan J. D. (1975). Influence on some rumen and blood parameters of feeding acetohydroxamic acid in a urea- containing ration for lambs. Can. J. Anim. Sci. 55, 39–47. 10.4141/cjas75-006

[B18] Jyothi N. Umamahe S. (2013). Production of protease and urease by kerosene utilizing fluorescent Pseudomonads isolated from local red latirite soil. Bioscan 8, 353–357.

[B19] Kakimoto S. Okazaki K. Sakane T. Imai K. Sumino Y. Akiyama S. I. (1989). Isolation and taxonomie characterization of acid urease-producing bacteria. Agric. Biol. Chem. 53, 1111–1117. 10.1080/00021369.1989.10869439

[B20] Kertz A. F. (2010). Review: urea feeding to dairy cattle: a historical perspective and review. Prof. Anim. Sci. 26, 257–272. 10.15232/s1080-7446(15)30593-3

[B21] Kilian M. (2015). Haemophilus, in Bergey's Manual of Systematics of Archaea and Bacteria, ed Whitman W. B. (New York, NY: Springer), 1–47. 10.1002/9781118960608.gbm01198

[B22] Kim J. N. Henriksen E. D. Cann I. K. Mackie R. I. (2014). Nitrogen utilization and metabolism in Ruminococcus albus 8. Appl. Environ. Microbiol. 80, 3095–3102. 10.1128/AEM.00029-14 24610852 PMC4018901

[B23] Kim M. Morrison M. Yu Z. (2011). Status of the phylogenetic diversity census of ruminal microbiomes. FEMS Microbiol. Ecol. 76, 49–63. 10.1111/j.1574-6941.2010.01029.x 21223325

[B24] Lozupone C. A. Hamady M. Kelley S. T. Knight R. (2007). Quantitative and qualitative beta diversity measures lead to different insights into factors that structure microbial communities. Appl. Environ. Microbiol. 73, 1576–1585. 10.1128/AEM.01996-06 17220268 PMC1828774

[B25] Lynd L. R. Weimer P. J. van Zyl W. H. Pretorius I. S. (2002). Microbial cellulose utilization: fundamentals and biotechnology. Microbiol. Mol. Biol. R 66, 506–577. 10.1128/MMBR.66.3.506-577.2002 12209002 PMC120791

[B26] Magoè T. Salzberg S. L. (2011). FLASH: fast length adjustment of short reads to improve genome assemblies. Bioinformatics 27, 2957–2963. 10.1093/bioinformatics/btr507 21903629 PMC3198573

[B27] Mahnert A. Moissl-Eichinger C. Berg G. (2015). Microbiome interplay: plants alter microbial abundance and diversity within the built environment. Front. Microbiol. 6:887. 10.3389/fmicb.2015.00887 26379656 PMC4552223

[B28] Makkar H. P. Sharma O. P. Dawra R. K. Negi S. S. (1981). Effect of acetohydroxamic acid on rumen urease activity *in vitro*. J. Dairy Sci. 64, 643–648. 10.3168/jds.S0022-0302(81)82624-0 7264024

[B29] Marri P. R. Paniscus M. Weyand N. J. Rendon M. A. Calton C. M. Hernandez D. R.. (2010). Genome sequencing reveals widespread virulence gene exchange among human Neisseria species. PLoS ONE 5:e11835. 10.1371/journal.pone.0011835 20676376 PMC2911385

[B30] McCrea K. W. Xie J. LaCross N. Patel M. Mukundan D. Murphy T. F.. (2008). Relationships of nontypeable *Haemophilus influenzae* strains to hemolytic and nonhemolytic *Haemophilus haemolyticus* strains. J. Clin. Microbiol. 46, 406–416. 10.1128/JCM.01832-07 18039799 PMC2238123

[B31] Minas K. McEwan N. R. Newbold C. J. Scott K. P. (2011). Optimization of a high-throughput CTAB-based protocol for the extraction of qPCR-grade DNA from rumen fluid, plant and bacterial pure cultures. FEMS Microbiol. Lett. 325, 162–169. 10.1111/j.1574-6968.2011.02424.x 22029887

[B32] Morou-Bermudez E. Burne R. A. (1999). Genetic and physiologic characterization of urease of *Actinomyces naeslundii*. Infect. Immun. 67, 504–512. 9916052 10.1128/iai.67.2.504-512.1999 PMC96348

[B33] Morou-Bermudez E. Burne R. A. (2000). Analysis of urease expression in *Actinomyces naeslundii* WVU45. Infect. Immun. 68, 6670–6676. 10.1128/IAI.68.12.6670-6676.2000 11083780 PMC97765

[B34] Nelson M. C. Morrison H. G. Benjamino J. Grim S. L. Graf J. (2014). Analysis, optimization and verification of Illumina-generated 16S rRNA gene amplicon surveys. PLoS ONE 9:e94249. 10.1371/journal.pone.0094249 24722003 PMC3983156

[B35] Oyeleke S. Okusanmi T. (2008). Isolation and characterization of cellulose hydrolysing microorganism from the rumen of ruminants. Afr. J. Biotechnol. 7, 1503–1504. 10.5897/AJB08.142

[B36] Pandya P. Singh K. Parnerkar S. Tripathi A. Mehta H. Rank D.. (2010). Bacterial diversity in the rumen of Indian Surti buffalo (*Bubalus bubalis*), assessed by 16S rDNA analysis. J. Appl. Genet. 51, 395–402. 10.1007/BF03208869 20720314

[B37] Patra A. K. (2015). Urea/Ammonia metabolism in the rumen and toxicity in ruminants, in Rumen Microbiology: From Evolution to Revolution, eds Puniya A. K. Singh R. Kamra D. N. (New Delhi; Heidelberg; New York, NY; Dordrecht; London: Springer), 329–341.

[B38] Pope P. Smith W. Denman S. Tringe S. Barry K. Hugenholtz P.. (2011). Isolation of Succinivibrionaceae implicated in low methane emissions from Tammar wallabies. Science 333, 646–648. 10.1126/science.1205760 21719642

[B39] Rasko D. A. Ravel J. Økstad O. A. Helgason E. Cer R. Z. Jiang L.. (2004). The genome sequence of *Bacillus cereus* ATCC 10987 reveals metabolic adaptations and a large plasmid related to *Bacillus anthracis* pXO1. Nucleic Acids Res. 32, 977–988. 10.1093/nar/gkh,258 14960714 PMC373394

[B40] R Core Team (2013). R: A Language and Environment for Statistical Computing. R Foundation for Statistical Computing Vienna Available online at: http://www.R-project.org/

[B41] Reed K. E. (2001). Restriction enzyme mapping of bacterial urease genes: using degenerate primers to expand experimental outcomes. Biochem. Mol. Biol. Educ. 29, 239–244. 10.1111/j.1539-3429.2001.tb00131.x

[B42] Sakai K. Yamauchi T. Nakasu F. Ohe T. (1996). Biodegradation of cellulose acetate by *Neisseria sicca*. Biosci. Biotechnol. Biochem. 60, 1617–1622. 10.1271/bbb.60.1617 8987659

[B43] Santos E. Thompson F. (2014). The Family Succinivibrionaceae, in The Prokaryotes, eds Dworkin M. Falkow S. Rosenberg E. Schleifer K. H. Stackebrandt E. (Berlin; Heidelberg: Springer), 639–648.

[B44] Sarda D. Choonia H. S. Sarode D. Lele S. (2009). Biocalcification by *Bacillus pasteurii* urease: a novel application. J. Ind. Microbiol. Biot. 36, 1111–1115. 10.1007/s10295-009-0581-4 19415357

[B45] Schaal K. P. Yassin A. A. (2015). Actinomyces, in Bergey's Manual of Systematics of Archaea and Bacteria, ed Whitman W. B. (New York, NY: Springer), 1–112.

[B46] Shen W. Jiang Y. Wang J. Bu D. Sun P. Jin E. (2012). Design and testing of rumen simulation system with discharging solid chime, liquid, and gas respectively. Trans. Chin. Soc. Agric. Eng. 28, 20–26. 10.3969/j.issn.1002-6819.2012.03.004

[B47] Singh B. K. Nunan N. Millard P. (2009). Response of fungal, bacterial and ureolytic communities to synthetic sheep urine deposition in a grassland soil. FEMS Microbiol. Ecol. 70, 109–117. 10.1111/j.1574-6941.2009.00731.x 19622069

[B48] Su J. Jin L. Jiang Q. Sun W. Zhang F. Li Z. (2013). Phylogenetically diverse *ureC* genes and their expression suggest the urea utilization by bacterial symbionts in marine sponge Xestospongia testudinaria. PLoS ONE 8:e64848. 10.1371/journal.pone.0064848 23741404 PMC3669342

[B49] Šul'ák M. Sikorová L. Jankuvová J. Javorskı P. Pristaš P. (2012). Variability of Actinobacteria, a minor component of rumen microflora. Folia Microbiol. 57, 351–353. 10.1007/s12223-012-0140-7 22528311

[B50] Upadhyay L. S. B. (2012). Urease inhibitors: a review. Indian J. Biotechnol. 11, 381–388.

[B51] Wang Q. Garrity G. M. Tiedje J. M. Cole J. R. (2007). Naive Bayesian classifier for rapid assignment of rRNA sequences into the new bacterial taxonomy. Appl. Environ. Microbiol. 73, 5261–5267. 10.1128/AEM.00062-07 17586664 PMC1950982

[B52] Weller R. A. Pilgrim A. F. (1974). Passage of protozoa and volatile fatty acids from the rumen of the sheep and from a continuous *in vitro* fermentation system. Br. J. Nutr. 32, 341–351. 10.1079/BJN19740087 4213614

[B53] Williams A. Withers S. E. (1983). *Bacillus* spp. in the rumen ecosystem. Hemicellulose depolymerases and glycoside hydrolases of *Bacillus* spp. and rumen isolates grown under anaerobic conditions. J. Appl. Microbiol. 55, 283–292. 10.1111/j.1365-2672.1983.tb01325.x

[B54] Wozny M. A. Bryant M. P. Holdeman L. V. Moore W. E. (1977). Urease assay and urease-producing species of anaerobes in the bovine rumen and human feces. Appl. Environ. Microbiol. 33, 1097–1104. 879770 10.1128/aem.33.5.1097-1104.1977 PMC170833

[B55] Wu S. Baldwin R. L. Li W. Li C. Connor E. E. Li R. W. (2012). The bacterial community composition of the bovine rumen detected using pyrosequencing of 16S rRNA *Genes*. Metagenomics 1, 1–11. 10.4303/mg/235571

[B56] Zhang R. Zhu W. Zhu W. Liu J. Mao S. (2014). Effect of dietary forage sources on rumen microbiota, rumen fermentation and biogenic amines in dairy cows. J. Sci. Food Agric. 94, 1886–1895. 10.1002/jsfa.6508 24375419

[B57] Zhang Y. G. Shan A. S. Bao J. (2001). Effect of hydroquinone on ruminal urease in the sheep and its inhibition kinetics *in vitro*. Asian Aust. J. Anim. Sci. 14, 1216–1220. 10.5713/ajas.2001.1216

[B58] Zhao S. Wang J. Zheng N. Bu D. Sun P. Yu Z. (2015). Reducing microbial ureolytic activity in the rumen by immunization against urease therein. BMC Vet. Res. 11:94. 10.1186/s12917-015-0409-6 25889568 PMC4404106

[B59] Zotta T. Ricciardi A. Rossano R. Parente E. (2008). Urease production by *Streptococcus thermophilus*. Food Microbiol. 25, 113–119. 10.1016/j.fm.2007.07.001 17993384

