# Supplementary material for: Insights into Abundant Rumen Ureolytic Bacterial Community Using Rumen Simulation System
Source: Front Microbiol. 2016 Jun 28;7:1006. doi: 10.3389/fmicb.2016.01006 (PMC4923134; doi:10.3389/fmicb.2016.01006)
Supplement: Supplementary file 1 [file DataSheet1.docx]

Supplementary Material

Insights into abundant rumen ureolytic bacterial community using rumen simulation system

Di Jin, Shengguo Zhao, Pengpeng Wang, Nan Zheng, Dengpan Bu, Yves Beckers and Jiaqi Wang^*^

*** Correspondence:** Jiaqi Wang: jiaqiwang@vip.163.com

# 1. Supplementary materials and methods

Quantitative PCR for bacterial urease gene and 16S rRNA gene

# 2. Supplementary Figures and Tables

Supplementary Figure S1 Appearance of the rumen simulation system used in this study.

Supplementary Figure S2 Standard curves generated from plasmid DNA containing *ureC* gene (A) and partial 16S rRNA gene (B).

Supplementary Figure S3 PCR products amplified using the universal bacterial primers 515F and 806R. M, 100bp ladder; 1-16, PCR products amplified using DNA samples as templates (U0_A0: 1, 2, 9 and 10; U0_A0.45: 3, 4, 11 and 12; U5_A0: 5, 6, 13 and 14; U5_A0.45: 7, 8, 15 and 16); CK, PCR product amplified using sterile water as template.

Supplementary Table S1 Composition and nutrient levels of basal diets (Dry matter based)

**Quantitative PCR for bacterial urease gene and 16S rRNA gene**

Standard curves for urease gene of rumen ureolytic bacteria and 16S rRNA gene of total bacteria were established respectively. PCR were performed using UreC-F/UreC-R or 338F/533R as primers respectively and rumen microbial DNA as templates. The amplicons were ligated into the pMD18-T Easy vector (TaKaRa, Dalian, China), and the recombinant plasmids were transformed into *E. coli* JM109 cells (TaKaRa, Dalian, China). Plasmids with bacterial *ureC* gene or 16S rRNA gene were used to build standard curves. The copy numbers of the plasmids were calculated and then the plasmids were serial diluted (1:10). The standard curves were generated using the diluted plasmids DNA.

The DNA quantification was performed in an iQ^TM^5 Multicolor Real-Time PCR Detection System (Bio-Rad, USA) using SYBR^®^ Premix Ex Taq™ II (Takara, Dalian). Each qPCR reaction (20 μL) included 10 μL 2 × SYBR Master Mix, 4 μL nuclease-free water, 0.8 μL each forward and reverse primer (10 μM) and 2 μL DNA template. PCR cycle parameters for *ureC* gene detection were as follows: 95 °C for 3 min, followed by 40 cycles at 95 °C for 15 s, 52 °C for 30 s and 72 °C for 30 s. PCR cycle parameters for detecting 16S rRNA genes were as follows: 95 °C for 3 min, followed by 40 cycles at 95 °C for 15 s, 60 °C for 30 s and 72 °C for 30 s. Melting curves were prepared for each PCR reaction by collecting fluorescence signal at every 0.5°C increment when the temperature ramped from 60°C to 95°C. Each sample was run in triplicate, and both standards and samples were assayed on the same qPCR plate. The qPCR reaction efficiencies with the degenerate primers ureC-F/R and 338F/533R were 106.5% and 100.7% respectively.


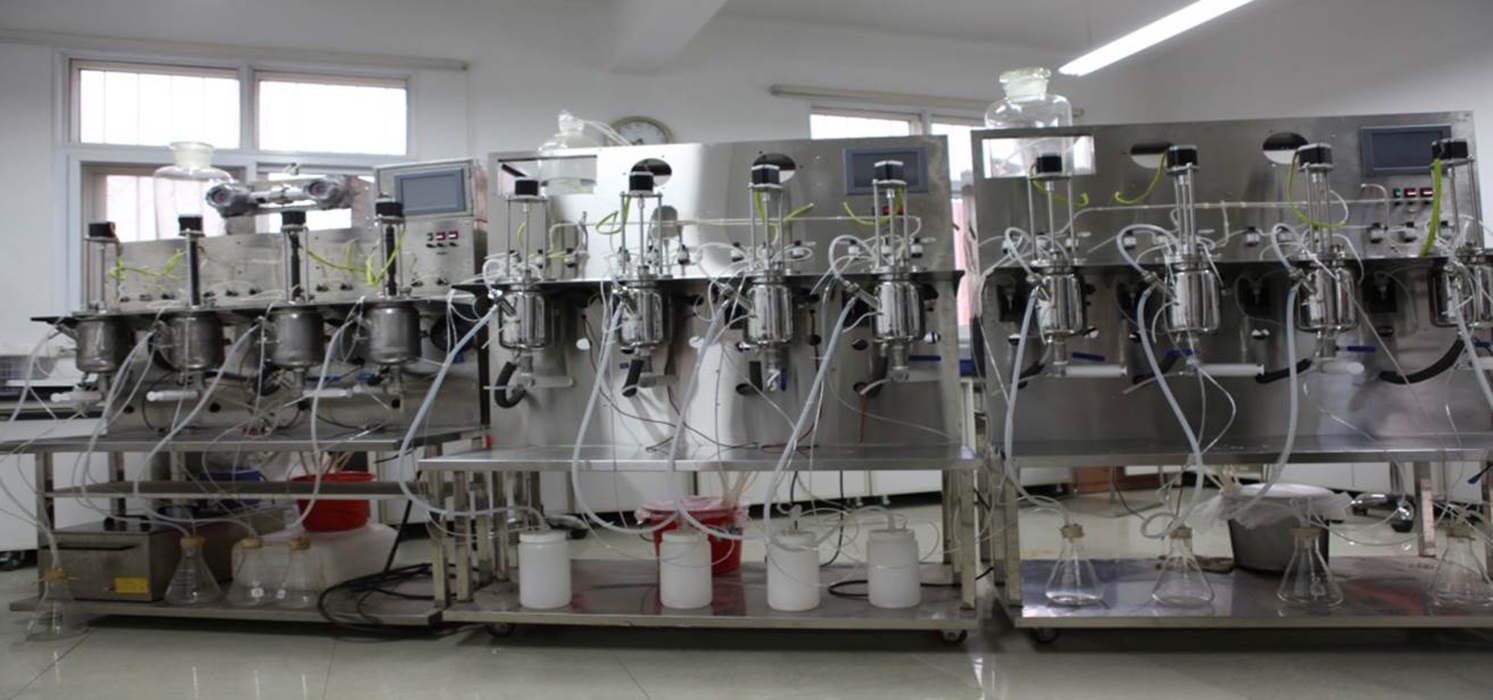


**Supplementary Figure S1** Appearance of the rumen stimulation system used in this study.





**Supplementary Figure S2** Standard curves generated from plasmid DNA containing *ureC* gene (A) and partial 16S rRNA gene (B).

**

**

**Supplementary Figure S3** PCR products amplified using the universal bacterial primers 515F and 806R. M, 100bp ladder; 1-16, PCR products amplified using DNA samples as templates (U0_A0: 1, 2, 9 and 10; U0_A0.45: 3, 4, 11 and 12; U5_A0: 5, 6, 13 and 14; U5_A0.45: 7, 8, 15 and 16); CK, PCR product amplified using sterile water as template.

**Supplementary Table S1** Composition and nutrient levels of basal diets (Dry matter based)

| **Item** | **Content (%)** |
| --- | --- |
| **Ingredients** |  |
| Alfalfa hay | 17.72 |
| Corn silage | 17.50 |
| Oaten hay | 5.09 |
| Cotton seed | 5.61 |
| Apple pulp | 3.74 |
| Sugar beet pulp | 6.71 |
| Molasses (30%) | 2.68 |
| Compound packet ^a^ | 40.95 |
| **Nutrient levels** |  |
| CP | 16.50 |
| NDF | 35.46 |
| ADF | 21.71 |
| EE | 6.46 |
| Ca | 0.97 |
| P | 0.35 |

^a^ The compound packet provided the following per kg of diets: Steam corn 180.39 g, Soybean skin 55.84 g, Soybean meal 64.43 g, Extruded soybean 38.66 g, DDGS 24.48 g, Double-low rapeseed meal 25.77 g, Ca(HCO_3_)_2_ 2.58 g, CaCO_3_ 2.58 g, NaCl 3.44 g, NaHCO_3_ 6.01 g.
